# Environmental enrichment through virtual reality as multisensory stimulation to mitigate the negative effects of prolonged bed rest

**DOI:** 10.3389/fnagi.2023.1169683

**Published:** 2023-08-22

**Authors:** Luka Šlosar, Manca Peskar, Rado Pišot, Uros Marusic

**Affiliations:** ^1^Science and Research Centre Koper, Institute for Kinesiology Research, Koper, Slovenia; ^2^Alma Mater Europaea – ECM, Department of Health Sciences, Maribor, Slovenia; ^3^Biological Psychology and Neuroergonomics, Department of Psychology and Ergonomics, Faculty V: Mechanical Engineering and Transport Systems, Technische Universität Berlin, Berlin, Germany

**Keywords:** physical inactivity, bed rest, disuse, mechanical unloading, non-physical interventions, virtual reality

## Abstract

Prolonged bed rest causes a multitude of deleterious physiological changes in the human body that require interventions even during immobilization to prevent or minimize these negative effects. In addition to other interventions such as physical and nutritional therapy, non-physical interventions such as cognitive training, motor imagery, and action observation have demonstrated efficacy in mitigating or improving not only cognitive but also motor outcomes in bedridden patients. Recent technological advances have opened new opportunities to implement such non-physical interventions in semi- or fully-immersive environments to enable the development of bed rest countermeasures. Extended Reality (XR), which covers augmented reality (AR), mixed reality (MR), and virtual reality (VR), can enhance the training process by further engaging the kinesthetic, visual, and auditory senses. XR-based enriched environments offer a promising research avenue to investigate the effects of multisensory stimulation on motor rehabilitation and to counteract dysfunctional brain mechanisms that occur during prolonged bed rest. This review discussed the use of enriched environment applications in bedridden patients as a promising tool to improve patient rehabilitation outcomes and suggested their integration into existing treatment protocols to improve patient care. Finally, the neurobiological mechanisms associated with the positive cognitive and motor effects of an enriched environment are highlighted.

## Introduction

Prolonged bed rest has been identified as a risk factor for physiological deconditioning since 1947. A seminal study entitled “The Dangers of Going to Bed,” (Asher, 1947) called attention to the risks it posed to older adults and the general population. Recent research conducted on hospitalized older adults has revealed that these patients spend up to 86% of their hospital days inactive, even though only a small percentage of cases, 5%, had a medical indication for bed rest (Jasper et al., 2020). This type of behavior is detrimental to both the physical and mental health of patients and poses a significant risk for functional independence and chronic disability, collectively referred to as hospital-associated disability (Loyd et al., 2020).

Recent advances in the field of aerospace science and the development of experimental models of forced bed rest in healthy subjects provided a better physiological understanding of immobilization and strategies to counteract immobilization-induced functional deterioration. Prolonged immobilization can lead to adverse consequences not only in older adults, but also in younger individuals, affecting cardiovascular (Hoffmann et al., 2022), endocrine (Belavy et al., 2012), immune (Hoff et al., 2015), gastrointestinal (Iovino et al., 2013), vestibular (Dyckman et al., 2012), and cognitive (Lipnicki et al., 2009) systems. An interesting phenomena of non-uniform loss of muscle mass and strength was recently systematically reviewed on 318 subjects exposed to experimental bed rest (Marusic et al., 2021). After longer periods of bed rest, such as 35 days, the decline in strength was found to be two times higher compared to muscle atrophy. In the early days of bed rest, such as 5 days, even higher ratios were reported (Marusic et al., 2021). These findings raise new questions about the underlying mechanisms responsible for the disproportionate decline in strength compared with muscle atrophy. They also highlight the importance of early interventions to prevent or minimize the adverse effects of prolonged bed rest.

Body posture and prolonged bed rest also directly affect the brain, which was mainly studied with electroencephalography (EEG) and functional magnetic resonance imaging (fMRI). One of the first reviews to examine EEG dynamics under bed rest conditions reported changes in the theta and alpha bands suggestive of cortical inhibition, and highlighted the need for further evidence in this area (Marušič et al., 2014). More specifically, the 6° head-down tilt position (HDTP) during the bed rest reduced the resting-state spectral power within the delta, theta, alpha, and beta frequency bands (Brauns et al., 2021b). Lower activity in alpha and beta frequency bands was also observed in several sources within the centroparietal and occipital regions. These effects occurred shortly after posture establishment, remained stable during 60 days of bed rest, and returned to baseline upon the end of the bed rest (Brauns et al., 2021b). In addition to posture-specific changes in brain activity, functional brain changes, such as decreased amplitudes of P300 and late positive potential (LPP) of the event-related potentials (ERPs) indicated that 30-day bed rest adversely affected affective picture processing suggesting that physical inactivity might play a role in emotion regulation. These effects were localized in the insula, precuneus, and cingulate gyrus (Brauns et al., 2019). Furthermore, the investigation into electrocortical correlates of selective attention showed that a 60-day bed rest negatively affected task performance and ERP potentials in fronto-central and parietal brain regions. Importantly, these data did not return to their baseline values after an eight-day recovery period (Brauns et al., 2021a). A preliminary data on eight bedridden healthy older adults showed increased P100 and P200 amplitudes and decreased P100 latencies after being exposed to 14 days of horizontal bed rest (Marušiè et al., 2021). Finally, Friedl-Werner et al. (2020) used fMRI to show impaired memory formation and associated dysfunctional mechanisms in the hippocampus and parahippocampus after 60 days of continuous bed rest. Taken together, these studies suggest that immobilization and inactivity resulting from prolonged bed rest induce functional brain changes and cognitive impairments, the recovery of which may be longer than the cessation of bed rest. To counteract the formation of dysfunctional brain mechanisms and cognitive impairment, appropriate intervention strategies must be implemented during bed rest as part of a comprehensive recovery strategy. The recovery process following prolonged bed rest deconditioning is a complex and multifactorial process influenced by several factors, including the duration of bed rest, age, overall health status, and the degree of deconditioning. In older adults, the detrimental effects of skeletal muscle deconditioning are particularly pronounced and may even lead to catabolic changes in muscle tissue that favor the development of sarcopenia, as shown in a recent meta-analysis (Di Girolamo et al., 2021). In addition to various countermeasures developed to alleviate the deleterious effects of prolonged immobilization, such as centrifugation (Kramer et al., 2020), nutritional support (Gao and Chilibeck, 2020), and aerobic interventions (Holt et al., 2016), non-physical rehabilitation interventions (Marusic and Grosprêtre, 2018) administered during immobilization resulted in significant improvements in cognitive (Marusic et al., 2018, 2019) as well as physical function (Marusic et al., 2015; Paravlic et al., 2018). Non-physical rehabilitation encompasses interventions that focus on cognitive and/or sensory stimulation to improve cognitive and physical function rather than physical exercise or movement. Interventions aimed at enhancing sensory stimulation include multiple modalities, including visual, auditory, and tactile stimulation, with the goal of promoting an engaging and interactive experience for the individual. Virtual reality (VR) as a form of enriched environment holds the potential of a breakthrough technology for non-physical rehabilitation by providing multisensory information and more realistic simulations to improve patient rehabilitation outcomes. This paper reviewed current non-physical rehabilitation practices, assessed the potential impact of integrating VR systems in enhancing the recovery process, and finally highlighted the implicated neurobiological mechanisms associated with beneficial cognitive, and motor effects of enriched environment exposure. The report provided a synthesis of existing empirical evidence and suggested future avenues for investigation in this field.

## Non-physical rehabilitation techniques

The frailty commonly experienced by bedridden patients poses a challenge to the implementation of conventional physical rehabilitation therapies in the early stages of hospitalization. The resulting deprivation of sensory input, including somatosensory and proprioceptive information, along with bed confinement, leads to rapid alterations in the organization of the sensorimotor system (Langer et al., 2012). These alterations revealed to have detrimental effects on postural balance and mobility (Koppelmans et al., 2017), movement duration and accuracy (Bassolino et al., 2012), tactile acuity (Lissek et al., 2009), and muscle properties (Clark et al., 2006). The decline in motor performance is attributed to the lack of feedback and feedforward mechanisms of motor control, which affects postural predictions and real-time movement adjustments (Scotto et al., 2020). To counteract immobilization-induced functional decline, non-physical rehabilitation methods such as cognitive interventions (CI), motor imagery (MI), action observation (AO), and their combination, provide a valuable compensatory strategy (Marusic and Grosprêtre, 2018). These types of interventions can create an enriched environment in which certain cognitive functions can be trained (Marusic and Grosprêtre, 2018) or even a neural resemblance to actual voluntary movement can be established (Fox et al., 2016; Grosprêtre et al., 2016).

The field of CI encompasses various approaches, such as cognitive stimulation, cognitive rehabilitation, and cognitive training, as described by Marusic and Grosprêtre (2018). Briefly, cognitive stimulation involves social and group cognitive activities, including discussions and therapeutic conversations, with the goal of improving social and cognitive functioning. Cognitive rehabilitation uses personalized programs to improve activities of daily living, with healthcare providers, patients, and families working together to achieve goals primarily by improving cognitive function. Cognitive training consists of personalized, guided exercises tailored to individual abilities to improve cognitive function and can be delivered in paper-pencil or computerized versions (Marusic and Grosprêtre, 2018). As for the effects of bed rest on cognitive function, the results are still controversial; some studies indicated a positive (facilitating) effect, while others showed the opposite (Lipnicki and Gunga, 2009). Although there is limited literature on cognitive interventions during bed rest (Marusic et al., 2019), it is reasonable to assume that cognition (such as working memory, selective attention, inhibition, and cognitive flexibility) forms the basis of any non-physical intervention.

MI in which movements are mentally rehearsed through a kinesthetic experience or a visual representation with an internal or external perspective (Decety, 1996) elicits intracortical and corticospinal modulations that attenuate the deleterious effects of immobilization (Rannaud Monany et al., 2022). The kinesthetic experience, i.e., imagining the sensation experienced during the action, showed to be more efficient in motor learning (Fontani et al., 2014), in gaining (Yao et al., 2013), and in maintaining muscle strength (Paravlic et al., 2018), thus being generally more successful in activating sensorimotor representations (Meugnot et al., 2015; Oldrati et al., 2021). At the neurological level, the use of kinesthetic imagery resulted in greater similarity of activated brain networks to actual motor execution compared to visual methods (Yang et al., 2021). The results may be attributed to the insufficient sensory information in visual MI, which negatively affects the individual’s ability to form a vivid and detailed representation of the movement. Studies employing a combination of AO and MI showed increased effectiveness in motor learning and rehabilitation outcomes, supporting to our hypothesis and demonstrating superiority over the use of each method individually (Eaves et al., 2016; Marusic and Grosprêtre, 2018). In this combined approach, the internally generated kinesthetic representations of an action are synchronized with the concurrent perception of the movement, augmenting the sensory experience of individuals through the integration of visual and auditory inputs, thereby enhancing the vividness of the MI task and leading to an increased sense of embodiment (Meers et al., 2020). With this in mind, the integration of enriched environments such as VR, which create the illusion of physical movement, has the potential to enhance the activation of motor-related brain regions. As a result, specific neural circuits are further activated, facilitating the desired neuroplastic adaptations (Slater, 2017).

## Enriched environments: a multisensory approach for enhanced rehabilitation

In everyday life, people are typically exposed to variety of multimodal experiences, from the sounds of nature to the sights of the surrounding environment. However, in a hospital setting, these experiences are often limited, leading to a more restricted sensory experience. In addition, patients’ attention may be disproportionately focused on their struggles, which may impair their ability to participate effectively in the rehabilitation process.

Despite the effectiveness of MI and AO in mitigating the loss of various physiological factors and facilitating motor recovery in bedridden patients, the implementation of these practices is generally limited to highly controlled and structured rehabilitation environments with limited variability and complexity compared with the unpredictable and dynamic nature of daily living. Failure to consider the impact of broader contextual factors, such as emotional and environmental influences, on real-world performance will limit the rehabilitation experience and may compromise the overall effectiveness of rehabilitation outcomes. XR-based environmental enrichment systems, in contrast, allow for the implementation of realistic scenarios engaging the patient’s sensorimotor system (Brugada-Ramentol et al., 2022) due to the enhanced simulation of the kinesthetic, visual, and auditory senses. Moreover, the three-dimensionality (3D) of VR showed to elicit stronger fronto-parietal activations compared to AO and its two-dimensional (2D) representations (Jastorff et al., 2016).

**FIGURE 1:**
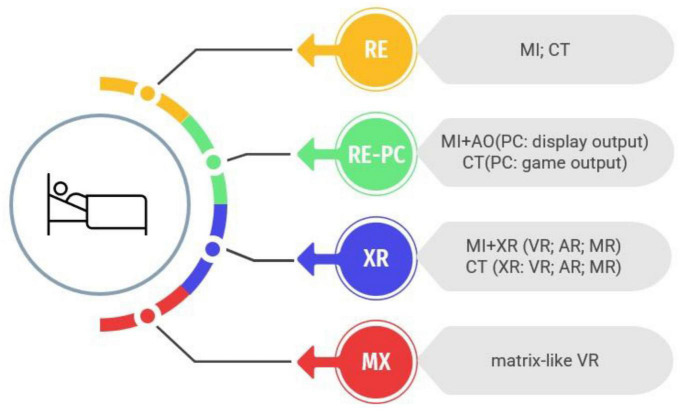
Evolution of intervention systems for bedridden patients: from real environment to fully immersive technologies. Intervention systems for bedridden patients have evolved over time, beginning with motor imagery (MI) and cognitive training (CT) in real environments (RE). These interventions have been enhanced with the use of personal computers and consoles (PC), incorporating action observation (AO) and displaying images through various screens. Advancements in technology have now made it possible to employ extended reality (XR) applications, such as virtual reality (VR), augmented reality (AR), and mixed reality (MR), in combination with MI or CT. Future developments aim to create fully immersive technologies that stimulate both the interoceptive and exteroceptive senses, a concept referred to as “Matrix-like” VR (MX).

Recent systematic reviews and meta-analyses have demonstrated the efficacy of VR in the rehabilitation of various conditions, including stroke (Leong et al., 2022), Parkinson’s disease (Kashif et al., 2022), and cerebral palsy (Ziab et al., 2022), with demonstrated functional improvements (Howard, 2017) and structural changes in the brain (Feitosa et al., 2022). However, according to Šlosar et al. (2022), a clearer terminology for the variety of digital environments (see Figure 1 for an overview) should be used to study the effects of interventions. Following this terminology, we proposed to use such non-physical interventions in conjunction with technological advances (Figure 1) in bedridden patients to mitigate the deterioration caused by bed rest.

## Personal computers and consoles with displays and controlling gadgets (PC)

In a 14-day bed rest study (Marusic et al., 2015), a computer-assisted spatial navigation intervention consisting of moving through virtual environments using a joystick controller was used to counteract the adverse effects of immobilization on gait performance in healthy older adults. Compared with the control group (passive watching of TV), the intervention group showed significant improvement in dual-task effects for self-selected and fast paced gait speed after bed rest. In the same study, control subjects were found to have increased gait variability under dual-task conditions (Marusic et al., 2015). The effects of such an intervention are explained in more detail in Marusic et al. (2019). In a usability study, Knols et al. (2017) demonstrated high acceptance and adherence to a gaming console adapted to be easily positioned at the patient bedside. Despite lacking clinical validation, the COPHYCON prototype showed significant short-term effects on measures of prefrontal cortex function in healthy elderly participants. In a study with patients with spinal cord injury (Villiger et al., 2013), an interactive game was integrated into an AO plus execution protocol. Wheelchair-bound participants were asked to observe an avatar performing movements with the lower limbs, and then mimic these movements by ankle flexion, hip extension, knee flexion, and leg adduction/abduction to control the avatar and complete gaming tasks. After a 4-week intervention period, assessments of gait capacity, postural stability, and muscle strength showed significant gains in lower extremity functionality. In addition, 50% of participants experienced reductions in both the intensity and unpleasantness of neuropathic pain symptoms. A study by Roosink et al. (2016) explored the use of a virtual feedback mechanism in a MI intervention for patients with the same pathology. The rehabilitation protocol consisted of performing MI of walking while seated in a wheelchair in front of a screen displaying an avatar walking through a forest. Participants were asked to concentrate on the sensory experiences produced by the interactive feedback, which was triggered by swinging their arms equipped with inertial sensors to match the pace of the imagined walking. The feasibility study reported improved vividness of MI with minimal adverse effects, indicating promising results for the response to MI interventions utilizing interactive feedback. Im et al. (2016) investigated the effects of combining MI (kinaestheic imagination of movements) with an interactive feedback mechanism on corticomotor excitability in both healthy older adults and stroke patients. They found that the combination resulted in increased amplitudes of motor evoked potentials compared to MI alone. This has significant implications for rehabilitation and recovery during periods of immobilization, as the combined approach can be utilized to target specific motor functions and improve motor performance, aiding in the recovery of lost motor abilities.

## Virtual reality (VR)

In contrast to PC-assisted interventions, VR systems allow users to experience a fully synthetic, computer-generated digital environment that replaces the physical world (Šlosar et al., 2022). The increased sense of embodiment that is perceived positively influences the user’s perception of their own body movements (Kong et al., 2017), leading to more accurate and effective outcomes in physical therapy. At the neurophysiological level, a study by Choi et al. (2020) demonstrated that event-related desynchronizations exhibited greater amplitudes with more distinct spatial features of the brain when MI is performed using a VR headset, compared to the display of the same images on a monitor. This modulation of neural activity by the degree of immersion provides important evidence for the use of VR technology in rehabilitation practice for bedridden patients (Xie et al., 2022). A recent study by Köyağasıoğlu et al. (2022) found that a 4-week intervention combining VR and MI significantly improved balance skills in healthy adults. While no significant differences were found in the center of pressure variable using a stabilometry device compared to a group combining PC and MI, the VR group demonstrated superior results on the Star Excursion Balance Test, particularly in posteromedial and posterolateral reach distances. In a similar experimental design, Bedir and Erhan (2021) compared the effects of 2D vs. 3D MI intervention on shot performances of archery, bowling, and curling athletes. Their findings showed the advantages of VR mental training in terms of shot performance after the 4-week training period. Yoshimura et al. (2020) investigated the effects of a VR contribution to the MI practice on the acquisition of prosthetic control using a prosthetic simulator in healthy individuals. Although the study was conducted with non-amputee participants, it yielded positive results in terms of supporting the daily activities of amputees, as evidenced by the enhancement in short-term prosthetic control acquisition following the acute practice of VR plus MI. In addition, self-assessed VR-based AO immersion level was found to have a negative correlation with the execution time of the bilateral manual dexterity task, supporting the idea that immersion is a crucial modulator of experience (Cummings and Bailenson, 2016) and thus has a positive influence on motor learning performance. See Table 1 for an overview of the potential benefits and distinctive features of different enriched environment approaches in long-term immobilization.

**TABLE 1:** Comparative overview of intervention approaches for enriched environment.

Intervention approach	Potential benefits	Distinctive features
Personal computers and consoles (PC)	Provides interactive and engaging activities to stimulate cognitive functions.	Familiar and widely accessible technology.
	Provides a diverse array of games and applications, fostering a stimulating environment.	Suitable for patients with varying levels of computer experience.
Virtual reality (VR)	Creates a fully immersive and interactive digital environment, enhancing sensory experiences.	Greater sense of immersion, leading to more effective outcomes in rehabilitation.
	Provides a more realistic and vivid experience, promoting a stronger sense of presence.	Potentially better neuro- physiological modulation during tasks due to the immersive nature.
	Allows for realistic simulations and scenarios for therapeutic purposes.	May facilitate improved body movement perception during virtual exercises.
	Offers a more stimulating and engaging atmosphere compared to traditional therapies.	Provides personalized and tailored programs with authentic scenarios for effective MI and AO practices.

**FIGURE 2:**
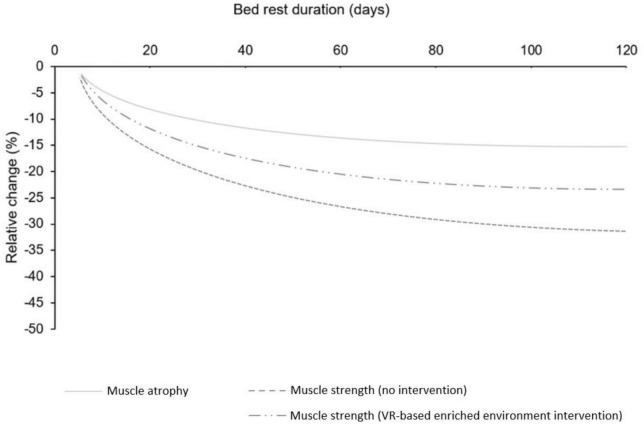
Speculative decline in muscle strength following XR intervention in conjunction with non-physical intervention: Adapted from Marusic et al. (2021).

In cases of functional decline due to immobilization, XR holds the potential to mitigate the early stages of muscle disuse-related declines in strength, which are attributed to loss of neuromuscular function (Campbell et al., 2019). The central and peripheral neural changes that occur can be effectively counteracted by corticospinal excitability elicited by MI in combination with VR. To illustrate the potential benefits of this approach, we adapted the figure from Marusic et al. (2021) showing the effects of bed rest on muscle atrophy and strength by adding a curve depicting the hypothetical decline in muscle strength if a XR intervention were implemented in conjunction with non-physical training interventions (Figure 2).

Despite the physical limitations imposed by illness or postoperative conditions that prevent patients from participating in conventional physical therapy, the psychosocial aspect of recovery is often overlooked. Previous research has found an association between the presence of anxiety and depressive symptoms and prolonged bed rest after discharge from critical care (Peris et al., 2011) and in experimental studies of bed rest (Ishizaki et al., 1994; Dimec Èasar, 2015). Enriched environments have been shown to be a critical tool in motivating patients to participate in rehabilitation practices (Boiko et al., 2022). They provide a stimulating and engaging atmosphere that promotes mental and emotional well-being, thus addressing patients’ often neglected psychosocial needs, resulting in better overall outcomes.

## Neurobiological mechanisms supporting beneficial effects of enriched environments

Several enriched environments related neurobiological mechanisms have thus far been recognized as neuroprotective and their effectiveness was also demonstrated in neurodegenerative disorders, such as in delaying the onset of Alzheimer’s disease (AD) (Liew et al., 2022) and the progression of Parkinson’s Disease (PD) (Alarcón et al., 2023). The following paragraphs highlight the most commonly known mechanisms, however, are not meant to provide an extensive overview (for this, see Liew et al., 2022; Alarcón et al., 2023).

Several animal studies have demonstrated that exposure to enriched environments led to beneficial effects on hippocampal structures, such as promoting hippocampal neurogenesis (Garthe et al., 2016), as well as increasing proliferation of progenitor cells and hippocampal cell survival (Olson et al., 2006; Ramírez-Rodríguez et al., 2014; Grońska-Pęski et al., 2021). Furthermore, the enriched environments exposure also restored the impaired neurogenesis in adult transgenic rodent models of AD after the deposition of Aβ plaques (Rodriguez et al., 2011; Valero et al., 2011; Llorens-Martín et al., 2013). The structural changes increasing the volume of the hippocampus may result in improved cognitive function (Hüttenrauch et al., 2016), while the hippocampal activity of the excitatory neurons could promote learning and memory formation (Stuchlik, 2014). Furthermore, enriched environments revealed to promote the expression of neurotrophins, such as the brain-derived neurotrophic factor (BDNF) (Kazlauckas et al., 2011; Kondo et al., 2012; Xu et al., 2015; Dandi et al., 2018), and nerve growth factors (NGF) (Torasdotter et al., 1998; Gelfo et al., 2011), which induce the differentiation and survival of neurons (Miranda et al., 2019), and regulate the excitatory and inhibitory transmission in the adult brain.

The dopaminergic system plays a central role in the pathology of PD and its dysfunction was implicated in movement and coordination difficulties (Glenthøj and Fibiger, 2019). Rodent models of PD mimic the neurodegeneration of the nigral dopaminergic system by inducing lesions and these studies have demonstrated that exposure to enriched environments beneficially affected the dopaminergic system including dopamine metabolism, the enzymes implicated in both dopamine synthesis and degradation, dopamine receptors, and its storage into vesicles (Jungling et al., 2017). The beneficial effects of enriched environments were also demonstrated in other neurotransmitter systems affected by PD, namely cholinergic, glutamatergic, and GABAergic (Alarcón et al., 2023).

Taken together, the neurobiological mechanisms supporting enriched environments, indicate that interventions combining sensory, cognitive, and physical stimulation at a heightened level could be used as a strategy for preventing cognitive/motor decline, but also as an approach or supporting treatment in managing aspects of complex neurodegenerative disorders.

## Conclusions and future perspectives

From a neurophysiological standpoint, observing movements promotes the development of motor skills (Ferrari, 1996). Among cutting-edge technologies, XR presents a viable way to activate the sensorimotor system and consequently boosting cognitive abilities and adaptability. XR in combination with MI can serve as a tool for enhanced sensorimotor feedback that promotes procedural learning. The use of 3D visualization systems that provide real-time 360-degree visual scanning can enhance the effectiveness of MI by allowing participants to rely on relevant stimuli and cues in a way that mimics real-world scenarios, thus overcoming the limitations of conventional 2D display methods used in AO. The enhanced proprioception, i.e., the sense of the position and movement of the body and its parts, and the vestibular system that arise from the user’s head movements while using a VR device (Michalski et al., 2019) provide a more interactive experience.

VR systems allow precise control of rehabilitation treatment, including manipulation of stimuli and distractors, so therapy sessions can be tailored to each individual’s needs. In this regard, the sensory information delivered through head-mounted displays goes beyond visual data, incorporating synchronized auditory information to further immerse the participant in the desired virtual environment. Current research focuses on incorporating haptic stimuli into VR-assisted MI to enhance the illusion of body ownership and the overall experience (Du et al., 2021). In addition, studies showed that the use of synchronized visual-haptic neurofeedback during MI can lead to improved outcomes in traditional neurofeedback training with brain-computer interfaces, particularly with respect to sensorimotor cortical activation (Wang et al., 2019).

Emerging evidence suggests that XR-based enriched environments may offer superior multisensory stimulation than traditional approaches, such as AO techniques combined with MI. However, the use of XR in bedridden patients is an area that requires further investigation, as most studies were limited to feasibility and usability assessments in symptomatic patients. In the absence of randomized controlled trials of the efficacy of XR in bedridden patients, it is difficult to draw definitive conclusions about its effectiveness. Future research is needed to fully understand the potential benefits and limitations of using XR-based enriched environments for bedridden patients and to explore how this technology can be integrated into existing treatment protocols to improve patient outcomes.

## Author contributions

UM, LŠ, and RP contributed to the initial idea and structure of the review. LŠ, UM, and MP contributed to the writing of the first draft of the manuscript. All authors contributed to the subsequent revisions and approved the final manuscript.
